# Efficacy and safety of olverembatinib as maintenance therapy after allogeneic hematopoietic cell transplantation in Philadelphia chromosome–positive acute lymphoblastic leukemia

**DOI:** 10.1007/s00277-025-06198-0

**Published:** 2025-01-16

**Authors:** Jun Kong, Feng-Mei Zheng, Chen-Hua Yan, Jing-Zhi Wang, Hai-Xia Fu, Zhi-Dong Wang, Pan Suo, Guan-Hua Hu, Meng Lv, Huan Chen, Xiao-Dong Mo, Lan-Ping Xu, Xiao-Hui Zhang, Xiao-Jun Huang, Yu Wang

**Affiliations:** 1 Beijing Key Laboratory of Hematopoietic Stem Cell Transplantation, Collaborative Innovation Center of Hematology, Peking University People’s Hospital, Peking University Institute of Hematology, National Clinical Research Center for Hematologic Disease, Peking University, Beijing, China; 2 Peking-Tsinghua Center for Life Sciences, Academy for Advanced Interdisciplinary Studies, Peking University, Beijing, China; 3 State Key Laboratory of Natural and Biomimetic Drugs, Peking University, Beijing, China; 4 Institute of Hematology, Peking University People’s Hospital, No. 11, Xizhimen South Street, Xicheng District, Beijing, 100044 People’s Republic of China

**Keywords:** Olverembatinib, Philadelphia chromosome–positive acute lymphoblastic leukemia, Allogeneic hematopoietic stem cell transplantation

## Abstract

Experience using olverembatinib as maintenance therapy in patients with Philadelphia chromosome–positive acute lymphoblastic leukemia (Ph^+^ ALL) after allogeneic hematopoietic cell transplantation (allo-HCT) is limited. We retrospectively collected data from 26 patients with Ph^+^ ALL who received only olverembatinib as maintenance therapy after allo-HCT. Olverembatinib was administered as prophylaxis in 18 patients (69.2%), and preemptively in 8 patients (30.8%). The median time of olverembatinib initiation after transplantation was 2.5 months (range, 1-7.3). The median starting dose of olverembatinib was 35 mg qod (range, 15–40). The median duration of olverembatinib treatment was 12.5 months (range, 6–23). Olverembatinib maintenance treatment was discontinued in 8 patients (8/26,30%), seven stopped the drug for a long-lasting *BCR-ABL1* negativity and 1 for recurrent fever associated with the drug. *BCR-ABL1* turned positive in 3 patients in 2, 3 and 6 months after discontinuation. During olverembatinib treatment, three patients developed grade ≥ 3 hematologic side effects, which resolved with dose interruption or dose reduction. The median follow-up time after allo-HCT were 17.75 months (range 7–31). The hematologic relapse rate was 7.7% (2/26), with no event in the preemptive group. The 3-year probability of overall survival and relapse free survival after allo-HCT was 91.7% and 79.1%, respectively. Only one patient in prophylaxis group died of central central nervous system (CNS) relapse. Thus, our data suggest that olverembatinib is effective and safe as maintenance treatment in patients with Ph^+^ ALL who underwent allo-HSCT. The main adverse effect was hematologic toxicity, which was tolerated.

## Introduction

Tyrosine kinase inhibitors (TKIs) combined with chemotherapy as frontline therapy and followed by allogeneic hematopoietic cell transplantation (allo-HCT) has improved the survival of patients with Philadelphia chromosome–positive acute lymphoblastic leukemia (Ph^+^ ALL) [1–3]. And maintenance therapy with first or second generation TKIs post-HCT in Ph + ALL can decrease relapse incidence and improve overall survival (OS) [3–9]. In two retrospective studies, the third-generation TKI ponatinib was evaluated safety and efficacy as prophylaxis and preemptive treatment with Ph^+^ ALL in the post-HCT setting [10, 11].

Olverembatinib, developed by Ascentage Pharma, is a new third-generation TKI designed to effectively target wild-type *BCR-ABL1* and a wide range of *BCR-ABL1* mutants, including *T315I*, making it a promising re-induction therapy for relapsed Ph^+^ ALL [12–14]. Olverembatinib was approved in China in November 2021 to treat adults with TKI-resistant chronic myeloid leukemia (CML) in the chronic or accelerated phase harboring the *T315I* mutation [15]. In November 2023, an additional indication was approved in China for adult patients with chronic phase CML resistant and/or intolerant of first- and second-generation TKIs. Data on adults with Ph^+^ ALL who were treated with olverembatinib have been reported in several small studies [16–18], but published experience regarding the use of olverembatinib in post-HCT settings is limited. In this study, we evaluated the use of olverembatinib in the post-HCT setting of patients with Ph^+^ ALL as maintenance treatment (including prophylaxis and preemptive treatment), focusing on its efficacy and safety.

## Methods

### Patient selection

We conducted a retrospective analysis to evaluate the efficacy and safety of olverembatinib as maintenance treatment (including prophylactic and preemptive treatment) in patients with Ph^+^ ALL after allo-HCT. The study was conducted in accordance with ethical tenets originating in the Declaration of Helsinki, and the protocol was reviewed and approved by the Ethics Committee of Peking University People’s Hospital. All patients provided informed consent for transplantation and the collection of clinical data.

All of the patients with Ph + ALL were recommended allo-HCT in our institution. The study’s inclusion criteria consisted of all of the following: (1) Ph + ALL undergoing allo-HCT, (2) administration of olverembatinib for prophylactic or preemptive purpose (no salvage), (3) duration of olverembatinib treatment > 3 months, (4) no others TKI post-allo-HCT and (5) allo-HCT for Ph + ALL between April 2022 and April 2024, regardless of conditioning regimen, donor type and graft source.

### TKI administration post-HCT in patients with Ph^+^ ALL

The prophylactic regimen refers to the initiation of TKIs consistent with pre-HCT TKIs use or according to the doctor’s choice in patients without measurable *BCR-ABL1* transcripts via quantitative polymerase chain reaction (qPCR) assessment after the first month post-HCT. The initiation time of prophylactic TKI occurred when the peripheral blood absolute neutrophil count was > 1.0 × 10^9^/L (without granulocyte-colony stimulating factor support), platelet count was > 50.0 × 10^9^/L, and measurable residual disease (MRD) was negative after HCT. Prophylactic therapy was scheduled for 12 to 24 months after transplantation.

Preemptive treatment was defined as therapy with TKIs when the *BCR-ABL1* transcript was measurable after HCT, irrespective of complete blood cell counts. If the patient had not initiated TKI therapy when *BCR-ABL1* was positive, the last effective TKI administered before transplantation was used or according to the doctor’s choice to initiate preemptive treatment. If *BCR-ABL1* changed from negative to positive during prophylactic TKI treatment, the TKI was changed to a higher-generation TKI for preemptive treatment.

The analysis endpoints were the cumulative incidence of hematologic relapse, relapse free survival (RFS) and overall survival (OS) after HCT, and the percentage of adverse events causing temporary or permanent suspension of treatment and/or dose reduction.

### Definitions of response to therapy and relapse

Bone marrow (BM) disease was assessed monthly via morphology, flow cytometry, and real-time qPCR (RT-qPCR), from treatment initiation until a major molecular response (MMR) was reached and then subsequently every 3 months. Complete remission (CR) was defined as < 5% BM blast cells and the absence of circulating blast cells and of extramedullary leukemia. MMR was defined by *BCR-ABL1/ABL1* ≤ 0.1% (3-log reduction from the standardized baseline); and complete molecular response (CMR) was defined as undetectable *BCR-ABL1* [12]. Molecular relapse after HCT was defined as the reappearance of *BCR-ABL1* expression in CMR patients.

Hematologic relapse was defined as ≥ 5% of BM or the presence of circulating blast cells. Central nervous system relapse was defined as the detection of blast cells based on morphology and flow cytometry of the cerebrospinal fluid. At the time of diagnosis, *ABL1* kinase domain mutation assays were not routinely performed. When patients had increased levels of *BCR-ABL1* transcripts after initial therapy or hematologic relapse at any time before or after HCT, Sanger direct sequencing was performed to identify mutations in the *BCR-ABL1* kinase domain [19, 20]. Adverse events (AEs) were assessed and graded according to the National Cancer Institute Common Toxicity Criteria, version 5.0 [21]. Acute and chronic graft-versus-host disease (GVHD) was graded according to the Mount Sinai Acute GVHD International Consortium (MAGIC) [22] and NIH criteria [23], respectively. Conditioning regimens and GVHD prophylaxis were administered as previously described [24–27].

### Statistical analysis

Descriptive analysis was performed using frequency distribution for continuous variables. The interval between the start date of olverembatinib therapy after transplantation and the date of the most recent visit was considered to be the follow up interval. For deceased patients, the date of the last follow-up was taken as the date of death. Survival analyses were performed using the Kaplan–Meier method and compared using the log-rank test. The confidence interval (CI) was 95%. OS was defined as continuous survival until death from any cause after HCT. OS values were calculated for the entire cohort of patients. Relapse-free survival (RFS) of the study population was defined as the time after allo-SCT until the hematologic relapse or death. Data analyses were performed using SPSS and R software packages (version 22.0 and 21.0, respectively), and *p* values < 0.05 were considered significant.

## Results

### Characteristics of patients and transplantation

We collected data from 26 patients with Ph^+^ ALL who had received allo-HCT and who were started olverembatinib after HCT between January 2022 and May 2024 at Peking University People’s Hospital, Peking University Institute of Hematology. The last follow up was in December 16,2024. Among the enrolled patients, four were under the age of 18 years. After HCT, all 26 patients achieved myeloid engraftment.

Among these patients, 18 were treated with prophylaxis, eight with preemptive treatment. The pre-HCT disease characteristics, last TKI before HCT, transplantation details, and incidence of GVHD are summarized in Table 1.

**Table 1:** Patient, disease, and treatment characteristics: pretransplant and transplantation

Category	Total, *n* = 26	Prophylaxis, *n* = 18	Preemptive, *n* = 8	*P* value
Gender ratio (Male/female), n	15/11	10/8	5/3	1.000
Age (yr) (< 18/≥18y<35y/≥35y)	4/7/15	2/6/10	2/1/5	0.442
WBC (×10^9^/L)(<30/≥30)	6/20	6/12	0/8	0.132
*BCR-ABL1* transcripts(P190/P210)	15/11	11/7	4/4	0.683
Disease status(CR1/≥CR2)	17/9	11/7	6/2	0.667
Pre-HCT CNSL, n(%)	3(11.5%)	3(16.7%)	0(0)	0.529
Pre-HCT T315I mutation, n(%)	7(26.9%)	6(33.3%)	1(12.5%)	0.375
Last TKI pre-HCT(2nd/3rd)	3/26	2/16	1/7	1.000
Pre-CAR-T therapy, n(%)	2(7.7%)	1(5.6%)	1(12.0%)	0.529
Pre-Blina/INO, n(%)	6(23.1%)	5(27.8%)	1(12.5%)	1.000
Pre-HCT *BCR-ABL1*				
unmeasurable/measurable	10/16	8/10	2/6	0.420
Donor type, n				
MSD/HID/MUD	5/19/2	2/15/1	3/4/1	0.375
Conditioning regimen				
BU based/TBI based	25/1	17/1	8/0	1.000
Acute GVHD, n(%)	9(34.6%)	6(33.3%)	3(37.5%)	1.000
Chronic GVHD, n(%)	5(19.2%)	2(11.1%)	3(37.5%)	0.281

Abbreviations: CR, complete remission; HCT, hematopoietic cell transplant; TKI, tyrosine kinase inhibitor; CNSL, central nervous system leukemia; CAR-T, chimeric antigen receptor T-cell therapy; Blina, blinatumomab; INO, inotuzumab ozogamicin; MSD, matched sibling donor; HID, haploidentical donor; MUD, matched unrelated donor; Bu, Busulfan; TBI, total body irradiation; GVHD, graft-versus-host disease

### Maintenance with olverembatinib after transplant and safety profiles

The median time of olverembatinib initiation after transplantation was 2.5 months (range, 1-7.3months). The median starting dose of olverembatinib was 35 mg qod (range, 15–40 mg). The median duration of olverembatinib treatment was 12.5 months (range, 6–23months). The main differences between prophylactic and preemptive olverembatinib maintenance strategy are reported in Table 2.

**Table 2:** Patient, disease, and treatment characteristics after HCT

Category	Total, *n* = 26	Prophylaxis, *n* = 18	Pre-emptive, *n* = 8	*P* value
Time to olverembatinib after HCT, median (range), month	2.5(1,7.3)	2.8(1,7.3)	1(1,4.5)	0.0590
Olverembatinib dosage, median (range), mg	35(15,40)	30(15,40)	40(20,40)	0.3350
Duration of olverembatinib therapy, median (range), month	12.5(6,23)	12(6,21)	16.25(6,23)	0.2870
Follow-up time, median (range), month	17.75(7,31)	16.75(7.5,31)	19(7,31)	0.7690
Patient outcome				
Relapse, n(%)	2(7.7%)	2(11.1%)	0(0)	
Hematologic, n(%)	1(3.8%)	1(5.6%)	0(0)	
CNS, n(%)	1(3.8%)	1(5.6%)	0(0)	
Discontinuation of olverembatinib, n(%)	8(30.8%)	5(27.8%)	3(37.5%)	
Time to discontinuation after HCT, median (range), month	17.75(9.5,24)	17.5(13,20.5)	23(9.5,24)	
Duration of olverembatinib therapy, median (range), month	15.25(6.5,22)	14.0(12,17)	22(6.5,22)	
*BCR-ABL1* turn to positve after discontinuation, n(%)	3(37.5%)	2(40.0%)	1(33.3%)	
Duration from discontinuation to being positive, median (range), month	3.0(2.0,6.0)			

Abbreviations: HCT, hematopoietic cell transplant; CNS, central nervous system

**Fig. 1:**
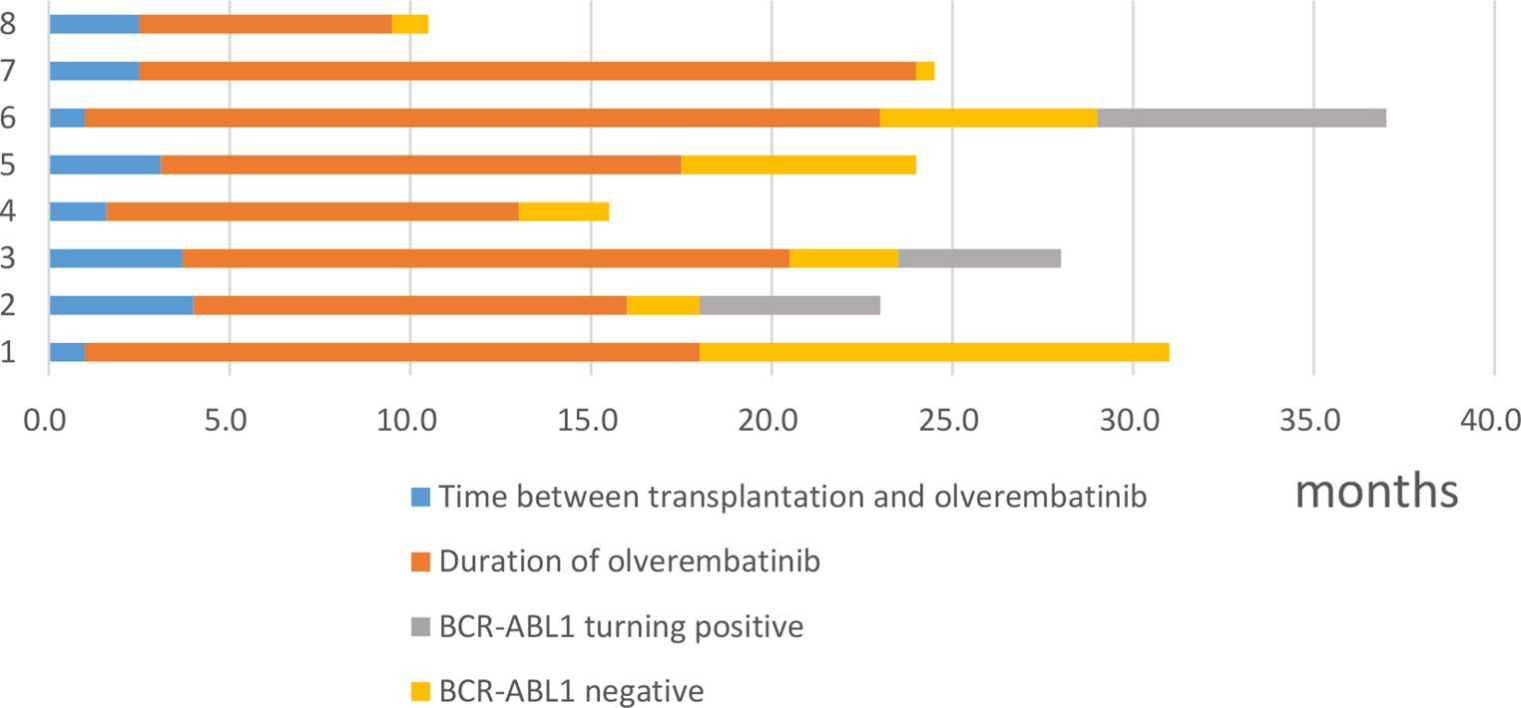
Patients who discontinued olverembatinib therapy after allo-HCT

Olverembatinib maintenance treatment was discontinued in 8 patients (8/26,30%), 5 in prophylaxis group and 3 in preemptive group. Among them, seven stopped the drug for a long-lasting MRD negativity (as per medical decision) and one for recurrent fever associated with the drug. *BCR-ABL1* turned positive in 3 patients in 2, 3 and 6 months after discontinuation of olverembatinib (Table 2; Fig. 1). And all the 3 patients restarted olverembatinib treatment.

Detailed data on the safety profile of olverembatinib are reported in Table 3. No deaths due to olverembatinib -related adverse events were reported. During olverembatinib treatment, three of the 26 patients developed grade ≥ 3 hematological side effects, including one patient with grade ≥ 3 neutropenia and two patients with grade ≥ 3 thrombocytopenia, which interrupted treatment. Common nonhematologic side effects of olverembatinib included grade 2 fever in 3 cases (11.5%), grade 2 hypertension in 3 cases (11.5%) and grade 1–2 head discomfort in 2 cases (7.7%). These adverse events were considered to be related to olverembatinib treatment. Olverembatinib-induced myelosuppression was reversed by dose interruption or dose reduction in all patients (Table 3). Only one patient discontinued olverembatinib treatment for recurrent fever after 6.5 months treatment.

**Table 3:** Side effects of olverematinib treatment

	Total, *n* = 26		Prophylaxis, *n* = 18		Preemptive, *n* = 8	
Event, n (%)	Any grades	G 3/4	Any grades	G 3/4	Any grades	G 3/4
Nonhematologic						
Fever	3(11.5%)	0(0)	2(11.1%)	0(0)	1(12.5%)	0(0)
Hypertension	3(11.5%)	0(0)	2(11.1%)	0(0)	1(12.5%)	0(0)
Head discomfort	2(7.7%)	0(0)	1(5.6%)	0(0)	1(12.5%)	0(0)
Hematologic						
Thrombocytopenia	9(34.6%)	2(7.7%)	4(22.2%)	2(11.1%)	5(62.5%)	0(0)
Anemia	7(26.9%)	0(0)	3(16.7%)	0(0)	4(20%)	0(0)
Leukopenia	6(23.1%)	1(3.8%)	5(27.8%)	1(5.6%)	1(12.5%)	0(0)

### Survival analysis

**Fig. 2:**
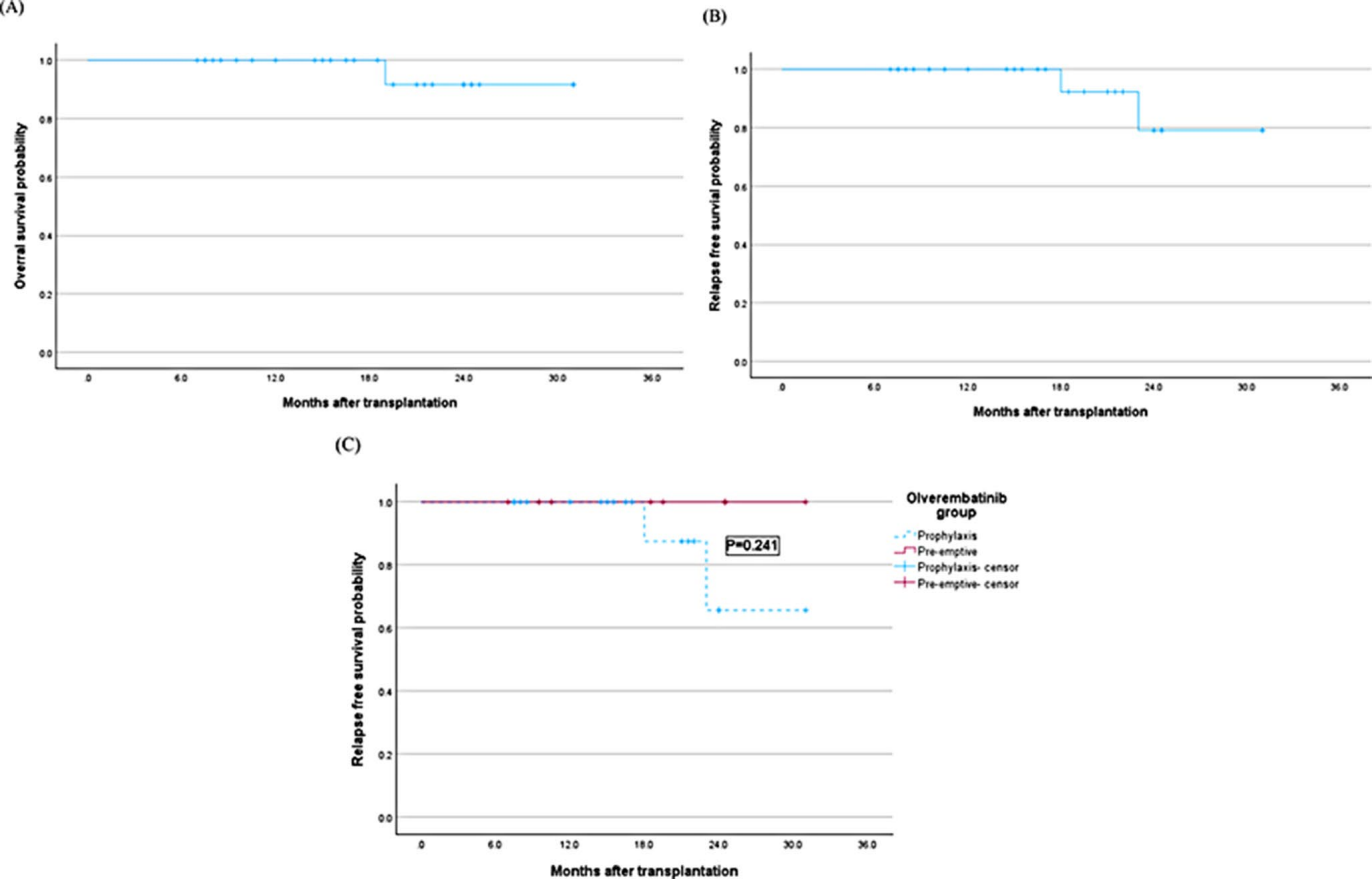
(**A**) Overall survival after allo-HCT (1 deaths in prophylactic group and 0 deaths in the preemptive group). (**B**) Relapse-free survival (RFS) after allo-HCT. (**C**) RFS according to the strategy (prophylaxis = dot line vs. pre-emptive)

The median follow-up time after allo-HCT were 17.75 months (range 7,31months). The hematologic relapse rate under olverembatinib maintenance therapy was 7.7% (2/26), with no event in the preemptive group and both the two events in the prophylactic group (*p* = 0.241). Among all the 26 patients, only one patient in prophylaxis group died (CNS relapse). The 3-year probability of OS and RFS after allo-HCT was 91.7% and 79.1%, respectively (Fig. 2A, B). The 3-year RFS after allo-HCT in prophylactic group was 65.6%, and was 100% in preemptively group (*p* = 0.241). The 3-year RFS after allo-HCT in patients with T315I mutation (7 patients) was 60%, and it was 100% in in patients without T315I mutation (19 patients) (*p* = 0.132).

## Discussion

New third-generation TKI, olverembatinib, exerts powerful inhibition of *BCR-ABL1* and has been approved to treat patients with CML that is resistant to or intolerant of second-generation TKIs and/or harbor *T315I* mutations. Olverembatinib is reported to be effective in patients with relapsed Ph^+^ ALL [16–18]. Herein, we report the efficacy and safety of olverembatinib as maintenance therapy in 26 patients with Ph^+^ ALL after allo-HCT.

In our study, patients had received only olverembatinib in the post-allo-HCT phase (no other TKIs), and the the therapeutic strategy was exclusively prophylactic (in 18/26 cases) or preemptive (in 8/26 cases); no cases were treated for hematologic relapse. The characteristics of our population receiving olverembatinib allow us to highlight some aspects that are worth reporting and that we believe may be useful in clinical practice.

Among the patients of our study, the 3-year probability of OS and RFS after allo-HCT was 91.7% and 79.1%, respectively. Anna Candoni et al. reported that the 5-year probability of OS and RFS after allo-HCT of pts who received ponatinib as maintenance therapy was 92% and 71%, respectively [28]. It seems that, as maintenance therapy, the efficacy of olverembatinib was similar to ponatinib. Of course, further research is needed to confirm this comparison.

In our study, RFS was similar between prophylactic group and preemptive group, and the initial time points of olverembatinib treatment were similar. Anna Candoni et al. reported that the RFS of prophylactic group was higher than preemptive group, and the ponatinib treatment started treatment earlier in prophylactic group than in preemptive group (4.3 months vs. 7.4 months, *p* = 0.01) [28]. Neeraj Saini et al. reported that the efficacy of TKI maintenance therapy started within the 3 months after transplantation was better than starting at 3 months after transplantation [6].

In patients receiving olverembatinib treatment after allo-HSCT, the most serious adverse effect of olverembatinib was myelosuppression. Thrombocytopenia had the highest incidence, which reached grade 3–4 in 33.3% of patients, but resolved after drug withdrawal. The main nonhematologic adverse reactions were hypertension and fever, which were relatively mild and tolerable. Reported rates of grade 3–4 myelosuppression caused by olverembatinib in CML treatment were 51.5% for thrombocytopenia, 23% for anemia, and 11.5% for neutropenia, which were higher than those observed in our study [15]. This may be related to the short duration of olverembatinib treatment in our study.

Our study had some limitations. First, this was a retrospective study, and some bias may have been present in the selection of patients who received olverembatinib. Second, the number of patients included was relatively small to evaluate the clinical efficacy of olverembatinib. Third, the observation period was insufficient to investigate the long-term efficacy and safety of olverembatinib. Further observational studies are required to answer these questions.

In conclusion, our data suggest that olverembatinib is effective as a prophylactic or preemptive treatment in patients with Ph^+^ ALL after allo-HCT. The main adverse effect was hematologic toxicity, which was generally tolerated.

## Data Availability

No datasets were generated or analysed during the current study.

## References

[1] Yanada M, Takeuchi J, Sugiura I, Akiyama H, Usui N, Yagasaki F, Kobayashi T, Ueda Y, Takeuchi M, Miyawaki S, Maruta A, Emi N, Miyazaki Y, Ohtake S, Jinnai I, Matsuo K, Naoe T, Ohno R, Japan Adult Leukemia Study Group (2006) High complete remission rate and promising outcome by combination of imatinib and chemotherapy for newly diagnosed BCR-ABL-positive acute lymphoblastic leukemia: a phase II study by the Japan Adult Leukemia Study Group. J Clin Oncol 24(3):460–466. 10.1200/JCO.2005.03.2177Epub 2005 Dec 12. PMID: 16344315 16344315 10.1200/JCO.2005.03.2177

[2] Tanguy-Schmidt A, Rousselot P, Chalandon Y, Cayuela JM, Hayette S, Vekemans MC, Escoffre M, Huguet F, Réa D, Delannoy A, Cahn JY, Vernant JP, Ifrah N, Dombret H, Thomas X (2013) Long-term follow-up of the imatinib GRAAPH-2003 study in newly diagnosed patients with de novo Philadelphia chromosome-positive acute lymphoblastic leukemia: a GRAALL study. Biol Blood Marrow Transpl 19(1):150–155. 10.1016/j.bbmt.2012.08.021Epub 2012 Sep 6. PMID: 22960387 10.1016/j.bbmt.2012.08.021 22960387

[3] Brissot E, Labopin M, Beckers MM, Socié G, Rambaldi A, Volin L, Finke J, Lenhoff S, Kröger N, Ossenkoppele GJ, Craddock CF, Yakoub-Agha I, Gürman G, Russell NH, Aljurf M, Potter MN, Nagler A, Ottmann O, Cornelissen JJ, Esteve J, Mohty M (2015) Tyrosine kinase inhibitors improve long-term outcome of allogeneic hematopoietic stem cell transplantation for adult patients with Philadelphia chromosome positive acute lymphoblastic leukemia. Haematologica 100(3):392–399. 10.3324/haematol.2014.116954Epub 2014 Dec 19. PMID: 25527562; PMCID: PMC4349279 25527562 10.3324/haematol.2014.116954 PMC4349279

[4] Chen H, Liu KY, Xu LP, Liu DH, Chen YH, Zhao XY, Han W, Zhang XH, Wang Y, Zhang YY, Qin YZ, Liu YR, Huang XJ (2012) Administration of imatinib after allogeneic hematopoietic stem cell transplantation may improve disease-free survival for patients with Philadelphia chromosome-positive acute lymphoblastic leukemia. J Hematol Oncol 5:29. 10.1186/1756-8722-5-29PMID: 22682059; PMCID: PMC3407007 22682059 10.1186/1756-8722-5-29 PMC3407007

[5] Nishiwaki S, Imai K, Mizuta S, Kanamori H, Ohashi K, Fukuda T, Onishi Y, Takahashi S, Uchida N, Eto T, Nakamae H, Yujiri T, Mori S, Nagamura-Inoue T, Suzuki R, Atsuta Y, Tanaka J (2016) Impact of MRD and TKI on allogeneic hematopoietic cell transplantation for Ph + ALL: a study from the adult ALL WG of the JSHCT. Bone Marrow Transpl 51(1):43–50. 10.1038/bmt.2015.217Epub 2015 Sep 21. PMID: 26389833 10.1038/bmt.2015.217 26389833

[6] Saini N, Marin D, Ledesma C, Delgado R, Rondon G, Popat UR, Bashir Q, Hosing CM, Nieto Y, Alousi AM, Qazilbash MH, Ciurea S, Shpall E, Khouri I, Kantarjian H, Jabbour E, Ravandi F, Champlin RE, Kebriaei P (2020) Impact of TKIs post-allogeneic hematopoietic cell transplantation in Philadelphia chromosome-positive ALL. Blood 136(15):1786–1789. 10.1182/blood.2019004685PMID: 32492706; PMCID: PMC8209550 32492706 10.1182/blood.2019004685 PMC8209550

[7] Hirschbühl K, Labopin M, Houhou M, Gabellier L, Labussière-Wallet H, Lioure B, Beelen D, Cornelissen J, Wulf G, Jindra P, Tilly H, Passweg J, Niittyvuopio R, Bug G, Schmid C, Nagler A, Giebel S, Mohty M (2021) Second- and third-generation tyrosine kinase inhibitors for Philadelphia-positive adult acute lymphoblastic leukemia relapsing post allogeneic stem cell transplantation-a registry study on behalf of the EBMT Acute Leukemia Working Party. Bone Marrow Transpl 56(5):1190–1199. 10.1038/s41409-020-01173-xEpub 2020 Dec 9. PMID: 33293597 10.1038/s41409-020-01173-x 33293597

[8] Wang Y, Chang YJ, Chen J, Han M, Hu J, Hu J, Huang H, Lai Y, Liu D, Liu Q, Luo Y, Jiang EL, Jiang M, Song Y, Tang XW, Wu D, Xia LH, Xu K, Zhang X, Zhang XH, Huang X (2024) Consensus on the monitoring, treatment, and prevention of leukaemia relapse after allogeneic haematopoietic stem cell transplantation in China: 2024 update. Cancer Lett.2024 Nov 28;605:217264. Epub ahead of print. PMID: 39332587 10.1016/j.canlet.2024.217264 39332587 10.1016/j.canlet.2024.217264

[9] Nakasone H (2023) Prophylactic or preemptive tyrosine kinase inhibitor therapy after allogeneic hematopoietic cell transplantation for Philadelphia chromosome-positive acute lymphoblastic leukemia. Int J Hematol 118(2):183–192. 10.1007/s12185-023-03556-4Epub 2023 Feb 20. PMID: 36807259 36807259 10.1007/s12185-023-03556-4

[10] Leotta S, Markovic U, Pirosa MC, Stella S, Tringali S, Martino M, Specchia G, Carluccio P, Risitano AM, Grimaldi F, Vigna E, Palmieri F, Palmieri R, Annunziata M, Pisapia G, Palazzo G, Milone GA, Pelle AC, Scalise L, Di Giorgio MA, Bulla A, Leotta V, Di Raimondo F, Milone G (2021) The role of ponatinib in adult BCR-ABL1 positive acute lymphoblastic leukemia after allogeneic transplantation: a real-life retrospective multicenter study. Ann Hematol 100(7):1743–1753. 10.1007/s00277-021-04504-0Epub 2021 Mar 28. PMID: 33774681 33774681 10.1007/s00277-021-04504-0

[11] Chen H, Xu LP, Zhang XH, Wang Y, Chen YH, Yan CH, Cheng YF, Han W, Chen Y, Qin YZ, Liu Y, Chang YJ, Liu KY, Huang XJ (2022) Safety and outcomes of maintenance therapy with third-generation tyrosine kinase inhibitor after allogeneic hematopoietic cell transplantation in Philadelphia chromosome positive acute lymphoblastic leukemia patients with T315I mutation. Leuk Res 121:106930. 10.1016/j.leukres.2022.106930Epub 2022 Aug 17. PMID: 36007342 36007342 10.1016/j.leukres.2022.106930

[12] Ren X, Pan X, Zhang Z, Wang D, Lu X, Li Y, Wen D, Long H, Luo J, Feng Y, Zhuang X, Zhang F, Liu J, Leng F, Lang X, Bai Y, She M, Tu Z, Pan J, Ding K (2013) Identification of GZD824 as an orally bioavailable inhibitor that targets phosphorylated and nonphosphorylated breakpoint cluster region-abelson (Bcr-Abl) kinase and overcomes clinically acquired mutation-induced resistance against imatinib. J Med Chem 56(3):879–894. 10.1021/jm301581yEpub 2013 Jan 28. PMID: 23301703 23301703 10.1021/jm301581y

[13] Ye W, Jiang Z, Lu X, Ren X, Deng M, Lin S, Xiao Y, Lin S, Wang S, Li B, Zheng Y, Lai P, Weng J, Wu D, Ma Y, Chen X, Wen Z, Chen Y, Feng X, Li Y, Liu P, Du X, Pei D, Yao Y, Xu B, Ding K, Li P (2016) GZD824 suppresses the growth of human B cell precursor acute lymphoblastic leukemia cells by inhibiting the SRC kinase and PI3K/AKT pathways. Oncotarget 8(50):87002–87015. 10.18632/oncotarget.10881PMID: 29152059; PMCID: PMC5675611 29152059 10.18632/oncotarget.10881 PMC5675611

[14] Wang Y, Zhang L, Tang X, Luo J, Tu Z, Jiang K, Ren X, Xu F, Chan S, Li Y, Zhang Z, Ding K (2020) GZD824 as a FLT3, FGFR1 and PDGFRα Inhibitor against Leukemia in Vitro and in vivo. Transl Oncol 13(4):100766 Epub 2020 Apr 1. PMID: 32247263; PMCID: PMC7125355 32247263 10.1016/j.tranon.2020.100766 PMC7125355

[15] Jiang Q, Li Z, Qin Y, Li W, Xu N, Liu B, Zhang Y, Meng L, Zhu H, Du X, Chen S, Liang Y, Hu Y, Liu X, Song Y, Men L, Chen Z, Niu Q, Wang H, Lu M, Yang D, Zhai Y, Huang X (2022) Olverembatinib (HQP1351), a well-tolerated and effective tyrosine kinase inhibitor for patients with T315I-mutated chronic myeloid leukemia: results of an open-label, multicenter phase 1/2 trial. J Hematol Oncol 15(1):113. 10.1186/s13045-022-01334-z. Erratum in: J Hematol Oncol. 2022;15(1):159. doi: 10.1186/s13045-022-01369-2. Erratum in: J Hematol Oncol. 2023;16(1):13. doi: 10.1186/s13045-023-01414-8. PMID: 35982483; PMCID: PMC9389804 10.1186/s13045-022-01334-z PMC9389804 35982483

[16] Liu C, Zhang X, Mao L, Qian J, Xiao F, Ye X, Wei J, Ye X, Jin J, Yu W (2023) Olverembatinib in relapsed Philadelphia chromosome-positive B-cell acute lymphoblastic leukemia: a study of 5 cases. Leuk Lymphoma 64(6):1208–1211 Epub 2023 Apr 17. PMID: 37067187 37067187 10.1080/10428194.2023.2197534

[17] Li X, Zhang J, Liu F, Liu T, Zhang R, Chen Y, Guo Y, Fang Y, Xu X, Pui CH, Zhu X (2023) Olverembatinib treatment in pediatric patients with relapsed philadelphia-chromosome-positive acute lymphoblastic leukemia. Clin Lymphoma Myeloma Leuk 23(9):660–666. doi: 10.1016/j.clml.2023.04.012. Epub 2023 May 11. PMID: 37301632 10.1016/j.clml.2023.04.012 37301632

[18] Zhu Y, Huang J, Wang Y, Han Y, Xue S, Yang Y, Zhu Y, Cai W, Chen S (2024) Olverembatinib treatment in adult patients with newly diagnosed Philadelphia chromosome-positive acute lymphoblastic leukemia. Ann Hematol 103(11):4643–4648. 10.1007/s00277-024-06027-wEpub 2024 Oct 14. PMID: 39400743 39400743 10.1007/s00277-024-06027-w

[19] Qin YZ, Liu YR, Zhu HH, Li JL, Ruan GR, Zhang Y, Jiang Q, Jiang H, Li LD, Chang Y, Huang XJ, Chen SS (2008) Different kinetic patterns of BCR-ABL transcript levels in imatinib-treated chronic myeloid leukemia patients after achieving complete cytogenetic response. Int J Lab Hematol 30(4):317– 23. 10.1111/j.1751-553X.2007.00962.x. PMID: 18665830 10.1111/j.1751-553X.2007.00962.x 18665830

[20] Qin Y, Chen S, Jiang B, Jiang Q, Jiang H, Li J, Li L, Lai Y, Liu Y, Huang X (2011) Characteristics of BCR-ABL kinase domain point mutations in Chinese imatinib-resistant chronic myeloid leukemia patients. Ann Hematol 90(1):47–52 Epub 2010 Aug 10. PMID: 20697894 20697894 10.1007/s00277-010-1039-5

[21] Common Terminology Criteria for Adverse Events (CTCAE) version 5. Published: November 27. US Department of Health and Human Services, National Institutes of Health, National Cancer Institute

[22] MacMillan ML, DeFor TE, Weisdorf DJ (2012) What predicts high risk acute graft-versus-host disease (GVHD) at onset? Identification of those at highest risk by a novel acute GVHD risk score. Br J Haematol 157(6):732–741. 10.1111/j.1365-2141.2012.09114.xEpub 2012 Apr 6. PMID: 22486355; PMCID: PMC3654854 22486355 10.1111/j.1365-2141.2012.09114.x PMC3654854

[23] Jagasia MH, Greinix HT, Arora M, Williams KM, Wolff D, Cowen EW, Palmer J, Weisdorf D, Treister NS, Cheng GS, Kerr H, Stratton P, Duarte RF, McDonald GB, Inamoto Y, Vigorito A, Arai S, Datiles MB, Jacobsohn D, Heller T, Kitko CL, Mitchell SA, Martin PJ, Shulman H, Wu RS, Cutler CS, Vogelsang GB, Lee SJ, Pavletic SZ, Flowers ME (2015) National Institutes of Health Consensus Development Project on Criteria for clinical trials in chronic graft-versus-host disease: I. The 2014 diagnosis and Staging Working Group report. Biol Blood Marrow Transpl 21(3):389–401e1 Epub 2014 Dec 18. PMID: 25529383; PMCID: PMC4329079 10.1016/j.bbmt.2014.12.001 PMC4329079 25529383

[24] Huang XJ, Liu DH, Liu KY, Xu LP, Chen H, Han W, Chen YH, Wang JZ, Gao ZY, Zhang YC, Jiang Q, Shi HX, Lu DP (2006) Haploidentical hematopoietic stem cell transplantation without in vitro T-cell depletion for the treatment of hematological malignancies. Bone Marrow Transplant 38(4):291-7. 10.1038/sj.bmt.1705445. Erratum in: Bone Marrow Transplant. 2008;42(4):295. Dosage error in article text. PMID: 16883312 10.1038/sj.bmt.1705445 16883312

[25] Chen H, Liu KY, Xu LP, Chen YH, Han W, Zhang XH, Wang Y, Qin YZ, Liu YR, Huang XJ (2015) Haploidentical hematopoietic stem cell transplantation without in vitro T cell depletion for the treatment of philadelphia chromosome-positive acute lymphoblastic leukemia. Biol Blood Marrow Transpl 21(6):1110–1116 Epub 2015 Feb 16. PMID: 25698612 10.1016/j.bbmt.2015.02.009 25698612

[26] Cheng C, Liang S, Yue K, Wu N, Li Z, Dong T, Dong X, Ling M, Jiang Q, Liu J, Huang XJ (2024) STAT5 is essential for inducing the suppressive subset and attenuate cytotoxicity of Vδ2 + T cells in acute myeloid leukemia. Cancer Lett 587:216730 Epub 2024 Feb 13. PMID: 38360140 38360140 10.1016/j.canlet.2024.216730

[27] Liang M, Lyu ZS, Zhang YY, Tang SQ, Xing T, Chen YH, Wang Y, Jiang Q, Xu LP, Zhang XH, Huang XJ, Kong Y (2024) Activation of PPARδ in bone marrow endothelial progenitor cells improves their hematopoiesis-supporting ability after myelosuppressive injury. Cancer Lett 592:216937. doi: 10.1016/j.canlet.2024.216937. Epub 2024 May 3. PMID: 38704134 10.1016/j.canlet.2024.216937 38704134

[28] Candoni A, Chiusolo P, Lazzarotto D, Sartor C, Dargenio M, Chiaretti S, Skert C, Giglio F, Trappolini S, Fracchiolla NS, Medici S, Bresciani P, Cuoghi A, Papayannidis C (2024) Ponatinib as a prophylactic or pre-emptive strategy to prevent Cytological Relapse after allogeneic stem cell transplantation in patients with Philadelphia chromosome-positive Acute Lymphoblastic Leukemia transplanted in complete Cytological Remission. Cancers (Basel) 16(11):2108. 10.3390/cancers16112108PMID: 38893226; PMCID: PMC11171293 38893226 10.3390/cancers16112108 PMC11171293

